# Methods for Assessment of Viability and Germination of *Plasmodiophora brassicae* Resting Spores

**DOI:** 10.3389/fmicb.2021.823051

**Published:** 2022-01-05

**Authors:** Yao Wang, Birger Koopmann, Andreas von Tiedemann

**Affiliations:** Division of Plant Pathology and Crop Protection, Department of Crop Sciences, Georg-August-University Göttingen, Göttingen, Germany

**Keywords:** clubroot, staining, CLSM (confocal laser scanning microscopy), germination process, germination rate

## Abstract

Clubroot caused by the obligate biotrophic parasite *Plasmodiophora brassicae* is a destructive soil borne disease of cruciferous crops. Resting spores of *P. brassicae* can survive in the soil for a long period without hosts or external stimulants. The viability and germination rate of resting spores are crucial factors of the inoculum potential in the field. The accurate assessment of viability and germination rate is the foundation to evaluate the effect of control methods. In this study, we evaluated several methods for the assessment of viability and germination rate of *P. brassicae* resting spores. Dual staining with calcofluor white-propidium iodide (CFW-PI) or single stain with Evans blue showed reliable accuracy in estimating viability. CFW-PI was capable of reliably determining the viability within 10 min, while Evans blue required overnight incubation to obtain accurate results. Due to DNA degradation of heat treatments, acetone was selected to evaluate the efficiency of propidium monoazide (PMA)–quantitative PCR (qPCR) used for the quantification of DNA from viable cells. The staining with 4,6-Diamidine-2-phenylindole dihydrochloride (DAPI) and the use of differential interference contrast microscopy were suitable for the determination of resting spore germination rates. The latter method also allowed recording individual germination states of spores. Alternatively, dual staining with CFW-Nile red was successfully used to assess the germination rate of resting spores with a lethal pre-treatment. This study evaluates and confirms the suitability of various microscopic and molecular genetic methods for the determination of viability and germination of *P. brassicae* resting spores. Such methods are required to study factors in the soil regulating survival, dormancy and germination of *P. brassicae* resting spores causing clubroot disease in Brassicaceae hosts and therefore are fundamental to develop novel strategies of control.

## Introduction

*Plasmodiophora brassicae* is an obligate phytopathogen that causes clubroot disease on cruciferous crops. Infected plants become stunted and wilt, while the roots are distorted and swollen, compromised in the uptake of water and nutrients. This disease leads to considerable losses in yield and quality. The longevity and persistence of *P. brassicae* resting spores also results in a reduction in land capital value and revenue when fields are infected. Due to the severe damage caused by *P. brassicae* and its widespread geographic distribution, a variety of control methods have been developed, such as soil liming (Tremblay et al., 2005), crop rotation (Friberg et al., 2006; Hwang et al., 2015; Peng et al., 2015), chemical treatments (Suzuki et al., 1995; Tanaka et al., 1999; Mitani et al., 2003), cultivation of resistant cultivars (Kuginuki et al., 1999; Piao et al., 2004; Hirai, 2006), and biocontrol (Lahlali et al., 2013; Lahlali and Peng, 2014). However, there are no approved pesticides against clubroot available in Europe. The efficacy of biocontrol treatments in the field is limited (Tremblay et al., 2005; Zhou et al., 2014; Yu et al., 2015). Most clubroot resistance sources are race-specific and have not been durable, since virulent clubroot isolates (e.g., pathotype 5×) have been found which overcome the race-specific clubroot resistance (Diederichsen et al., 2009; Rahman et al., 2014). The development of novel, more effective strategies of control is hindered by a lack of accurate methods to study crucial stages in the life cycle of *P. brassicae* in the soil prior to infection.

**FIGURE 1:**
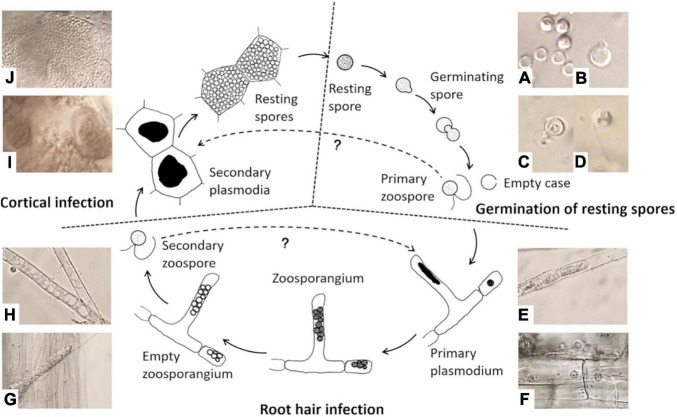
Life cycle of *Plasmodiophora brassicae*. **(A)** Resting spores (full) and germinated spores (empty); **(B)** emerged “papilla”; **(C)** germinating spore; **(D)** primary zoospore; **(E)** primary plasmodium in root hair; **(F)** primary plasmodium in epidermal cells; **(G)** zoosporangial cluster in root hair; **(H)** empty zoosporangium; **(I)** secondary plasmodia in cortical cells; **(J)** resting spores in cortical cells.

There are three stages in the life cycle of *P. brassicae*, survival in the soil as resting spores, root hair infection, and cortical invasion (Figure 1). *P. brassicae* can survive for a long period in the soil as resting spores. This is the only stage in which the pathogen can survive in the absence of host plants. Under favorable conditions, resting spores are induced to germinate and release primary biflagellate zoospores, which are required for infection in the field. Thus, the regulation of resting spore germination is a crucial factor for the completion of the pathogen life cycle through infection and disease induction. The poor knowledge on the nature and function of such regulatory factors–to a significant extend–is due to a lack of accurate and reliable methods to study spore activities. Accurate methods for assessing resting spore viability and germination are therefore essential in any studies aiming at the development of effective measures of disease control. However, such methods have not been systematically explored and evaluated and thus are lacking so far.

Several studies have examined the viability of resting spores by fluorescent dyes, such as the cellulose and chitin binding fluorochrome calcofluor white and the nucleic acid stain ethidium bromide (Takahashi and Yamaguchi, 1988). The azo dye Evans blue has been widely used in former studies to differentiate between dead and living cells as it can penetrate ruptured or destabilized membranes resulting in a blue-stained cytoplasm in dead cells, while living cells maintaining membrane integrity stay colorless (Jacyn Baker and Mock, 1994; Crutchfield et al., 1999; Lu and Higgins, 1999). However, Evans blue was shown not to be reliable in the estimation of *P. brassicae* resting spore viability, a longer exposure time (8 h or more) could improve its accuracy (Harding et al., 2019). Moreover, a cell membrane impermeable dye propidium monoazide (PMA) was employed to quantify the amount of viable spores by quantitative PCR (qPCR) instead of total DNA (Al-Daoud et al., 2017). PMA inhibits PCR amplification of DNA from dead cells by removing modified DNA during purification and inhibiting amplification by DNA polymerases (Nocker et al., 2006). For the determination of germinated resting spores, various methods have been tested to identify the absence of nuclei, including orcein (Naiki et al., 1987; Feng et al., 2010; Rashid et al., 2013), 4′-6-diamidino-2-phenylindole-dihydrochloride (DAPI; Niwa et al., 2008; Chen et al., 2018), and SYTO 82 orange fluorescent nucleic-acid stain (Niwa et al., 2008). Differential interference contrast (DIC) microscopy is a relatively simple method, which has been applied in many studies to distinguish germinated from non-germinated spores (Naiki et al., 1987; Takahashi, 1991; Suzuki et al., 1992; Asano et al., 2000). Currently, there is no systematic evaluation of these methods in order to provide suitable tools for studying *P. brassicae*.

In this study, several methods for the assessment of viability and germination of *P. brassicae* resting spores were examined and improved. Propidium iodide was used to replace the toxic dye ethidium bromide in the dual staining and its accuracy in estimating the viability was compared with Evans blue. For the evaluation of PMA qPCR, alternative methods were evaluated to inactivate the spores since heat treatments may cause DNA damage affecting the efficiency of PMA qPCR to assess the number of viable spores. Furthermore, three methods were examined to differentiate the germinated spores from non-germinated spores, including the nucleic stain DAPI, dual staining with calcofluor white-Nile red, as well as DIC microscopy.

## Materials and Methods

### Preparation of Spore Suspension

The single spore isolate H1 (provided by Prof. Elke Diederichsen, Freie Universität Berlin) was propagated on susceptible Chinese cabbage *B. rapa*. cv. Granaat in the greenhouse. The root galls were harvested 8 weeks post-inoculation (wpi) and stored at −20°C after rinsing. Resting spores were isolated from the root galls. The galls were surface-sterilized with 70% ethanol for 10 min, followed by 1% sodium hypochlorite for 10 min and then rinsed three times with sdH_2_O. The surface-sterilized galls were cut into small pieces and homogenized with sdH_2_O using an autoclaved homogenizer (Ultra-Turrax T25, IKA^®^-Werke GmbH & Co., KG, Germany). The homogenate was filtered through eight layers of sterile 50 μm nylon mesh, and the filtrate was centrifuged at 500 rpm for 10 min. The supernatant was subsequently transferred into new tubes and centrifuged at 3,500 rpm for 10 min to obtain the spore pellet. After rinsing twice, the pellet was resuspended with sdH_2_O. The concentration was determined using a hemocytometer and adjusted to 1.5 × 10^8^ spores/ml.

### Assessment of Resting Spore Viability

The freshly prepared spore suspension (100% viability) and the autoclaved spore suspension (considered as 100% mortality) were mixed at different ratios to obtain the assumed percentages of viable spores (0, 25, 50, 75, and 100%). This dilution series was used as standard to evaluate the performance of methods for assessing the viability of resting spores. To determine the optimal staining time, the resting spores were examined after exposure to the staining solution for 0, 0.25, 0.5, 2, 4, 24, 48, and 72 h at room temperature.

#### Evans Blue Staining

Evans blue (Merck KGaA, Germany) was applied to distinguish viable from non-viable resting spores. In a 1.5 ml microcentrifuge tube, 500 μl spore suspension was mixed with an equal volume of fresh Evans blue stain solution (20 mg/ml) and incubated at room temperature. Approximately 10 μl of each sample was placed on a slide and examined under a light microscope (Standard 20, Zeiss, Germany) at 400 × magnification.

#### Calcofluor White–Propidium Iodide Dual Staining

Calcofluor white solution (CFW, Merck KGaA, Germany) consisting of 1 g/l Calcofluor White M2R and 0.5 g/l Evans blue was used for the staining of resting spore cell walls. The stock solution of propidium iodide (PI, Carl Roth GmbH & Co., KG, Germany) at 1 mg/ml was used to intercalate with nucleic acids. An aliquot of 500 μl spore suspension was mixed with 50 μl CFW solution and 1 μl PI stock solution and incubated at room temperature in the dark. The excitation and emission wavelength (EX, EM) of dual staining were optimized. CFW was excited using a 405 nm laser and collected at 480–510 nm. The fluorescence of PI was excited at 514 and 561 nm, and emission was recorded at 600–610 nm. Confocal laser scanning microscopy (CLSM) was performed with a Leica TCS SP5 Laser Scanning Microscope 510 (Leica Microsystems CMS GmbH, Germany). Images were processed using the Leica Application Suite Advanced Fluorescence software (Version 2.6.3.8173).

#### Propidium Monoazide Treatment and Quantitative PCR

Resting spores exposed to different lethal treatments were incubated with cell membrane impermeable dye PMA, which inhibits amplification of DNA from dead spores, to examine the efficacy of PMA qPCR in quantifying DNA from viable spores.

##### Lethal Treatment

Resting spores were inactivated by different methods, examined for their effects on DNA integrity. For chemical treatments, spore suspension was incubated with an equal volume of HPLC grade methanol and acetone for 30 min, respectively. Sterile deionized water was used as control treatment. For heat treatments, spore suspension was subjected to 80°C-1 h in a water bath, 95°C-10 min in the digital dry bath heat block (NIPPON Genetics Europe, Germany) and 121°C-20 min in the autoclave. The viability of treated spores was examined using the dual staining with CFW-PI immediately and Evans blue for overnight incubation.

##### Propidium Monoazide Treatment

After lethal treatment, 300 μl of spore suspension was divided into three equal parts. One part was assessed for viability by the dual staining with CFW-PI. The other two parts were treated with 2.56 μl of 2 mM PMA (Biotium, United States) or an equal volume of sterile deionized water. The tubes were incubated in the dark on an orbital shaker at 300 rpm for 30 min at room temperature. Subsequently, the tubes were placed in a transparent box wrapped with a layer of aluminum foil and filled with ice. The samples were exposed to 500 W halogen light for 15 min at a distance of 20 cm and kept at continuous mixing on a shaker. After treatment, the samples were spun at 10,000 rpm to pellet the cells and sniped in liquid nitrogen for DNA extraction.

##### DNA Extraction

The samples were ground with 4 mm stainless beads into powder. Total DNA was extracted by a cetyltrimethylammonium bromide (CTAB) based method (Brandfass and Karlovsky, 2008). Briefly, the sample powder in each 2 ml tube was suspended in 1 ml CTAB solution with 1 μl proteinase K (20 mg/ml) and 2 μl β-mercaptoethanol and mixed well. The samples were then incubated at 42°C for 10 min, followed by 65°C for 10 min. Subsequently, the samples were purified by chloroform/isoamyl alcohol (24:1, v/v) extraction. DNA was precipitated by a mixture of 30% polyethylene glycol (PEG) and 5 M NaCl. Pelleted DNA was washed twice with cold 70% ethanol, allowed to dry in a speed vacuum concentrator, and resuspended in 200 μl of sterile Tris-EDTA (TE) buffer.

##### Quantitative PCR Detection

DNA of *P. brassicae* was amplified and quantified by a CFX384 real-time PCR instrument (Bio-Rad, Germany) using the primer pair CRqF2 (CTAGCGCTGCATCCCATATC) and CRqR2 (TGTTTCGGCTAGGATGGTTC) (Harding et al., 2019). A 10 μl PCR reaction consisted of 5 μl premix (qPCRBIO SyGreen Mix Lo-Rox, Nippon Genetics Europe GmbH), 0.4 μM of each primer and 1 μl DNA template and filled up with ddH_2_O. The qPCR program was one cycle of 2 min initial denaturation at 95°C followed by 40 cycles at 94°C for 15 s and 60°C for 30 s, and a cycle of 7 min at 72°C for final elongation.

##### Accuracy of the Propidium Monoazide Quantitative PCR Assessment

To verify the accuracy of the PMA qPCR, an acetone treatment was applied for cell inactivation. Half portion of the inactivated standard series suspension was treated with PMA, another portion was treated with equal volume of ddH_2_O. DNA was extracted as mentioned above. As a DNA-reference, plasmid pPK2II (ca.10,505 bp) was constructed by aqua cloning (Beyer et al., 2015) the rRNA cistron into blunt ended pUC19 with upstream primer sA1n (TACCTGGTTGATCCTGCCAGT) and downstream primer nH2r (GAATCAACGGTTCCTCTCGTACT) (Schwelm et al., 2016). The isolated plasmid DNA was quantified by the Qubit Fluorimeter (Invitrogen, Carlsbad, CA, United States) and accordingly diluted from 10 to 100,000 times. The copy number was calculated using the following equation (Whelan et al., 2003):


$$\mathrm{copy}\,\mathrm{number}=\frac{6.02\times 10^{23}\,\left(\mathrm{copy}/\mathrm{mol}\right)\times \mathrm{DNA}\,\mathrm{amount}\,\left(g\right)}{\mathrm{DNA}\,\mathrm{length}\,\left(\mathrm{bp}\right)\times 660\,\left(\left(g/\mathrm{mol}\right)/\mathrm{bp}\right)}$$

A standard curve was generated by plotting the logarithm of the template copy number against the corresponding CT value. The number of viable and total spores in each sample could be calculated by relating the CT value to the standard curve in order to obtain the viability.

### Determination of Resting Spore Germination Rate

#### 4′-6-Diamidino-2-Phenylindole-Dihydrochloride Staining

A nucleic acid stain, 4,6-Diamidine-2-phenylindole dihydrochloride (DAPI, Sigma-Aldrich, Germany), was used in a working solution of 1 μg/ml. After 5 min incubation at room temperature, an excitation wavelength of 405 nm and an emission wavelength of 430—550 nm was applied to observe the nuclei in non-germinated spores.

#### Calcofluor White–Nile Red Dual Staining

Nile red (9-diethylamino-5H-benzo (alpha) phenoxazine-5-one) (Sigma–Aldrich, Germany) was used for the detection of intracellular lipid droplets. It was dissolved in methanol to make a stock solution of 1 mg/ml. The concentration of working solution was 1 μg/ml. The resting spores were incubated with CFW and Nile red for 10 min in the dark. The signal of CFW was recorded as mentioned above. Lipid droplets stained by Nile red were detected with an excitation wavelength of 488 nm and an emission wavelength of 590–620 nm.

#### Differential Interference Contrast Microscopy

A Zeiss light microscope (Standard 20) with a DIC prism was used to differentiate germinated from non-germinated spores according to the description by Suzuki et al. (1992). Images produced by the DIC microscope were relief-like and seemed to have a shadow cast.

### Statistical Analysis

Statistical analyses were carried out with SPSS (version 26, IBM) and R (version 4.0.2). A Mixed-model Analyses of Variance (ANOVA) was performed using a General Linear Model (GLM) to determine significant differences for main factors and interactions between factors. Subsequently, Tukey’s Multiple Comparisons Tests were conducted to determine the statistically significant differences of means within each main factor.

## Results

### Viability Assessment by Staining

**FIGURE 2:**
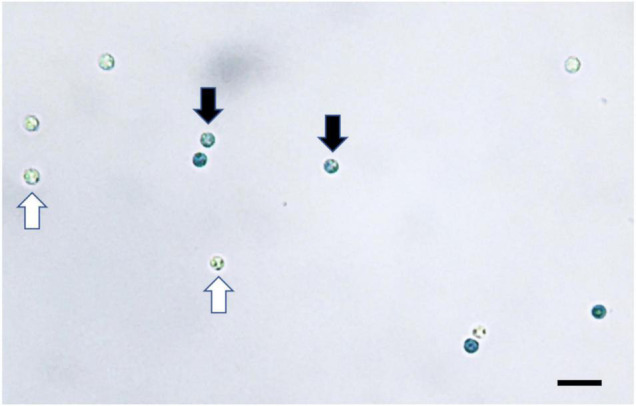
*Plasmodiophora brassicae* resting spores stained with Evans blue visualized under the optical microscope. Black arrows indicate dead spores (blue), white arrows indicate viable spores (colorless), bar = 10 μm.

**FIGURE 3:**
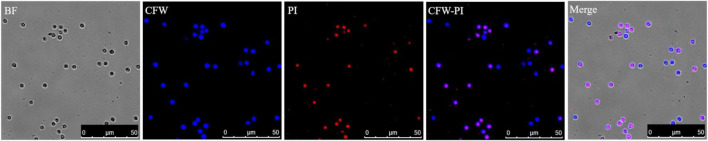
Confocal laser scanning microscopy (CLSM) images of *Plasmodiophora brassicae* resting spores double stained with calcofluor white (CFW)–propidium iodide (PI). Both dead and viable spores appear blue after CFW staining, while the nuclei of dead spores appear red after PI staining. In the merged image, the blue spores indicate viability and the pink spores are dead. BF, bright field.

Both staining methods of Evans blue and CFW-PI showed good performance to distinguish viable from non-viable spores. For Evans blue, viable spores remained colorless, while non-viable resting spores appeared in blue color due to the uptake of stain into the cytoplasm (Figure 2). For dual staining with CFW-PI, CFW staining alone only exhibited blue fluorescence, while PI staining alone produced detectable red fluorescence, indicating that each fluorescence was specific to each dye without the interference of autofluorescence. After configuration optimizations, the images of CFW-PI staining showed a clear differentiation between viable and dead spores (Figure 3). The cell wall of both dead and viable spores was stained by CFW showing blue color. The nucleic DNA of dead spores were intercalated with PI resulting in red color.

**FIGURE 4:**
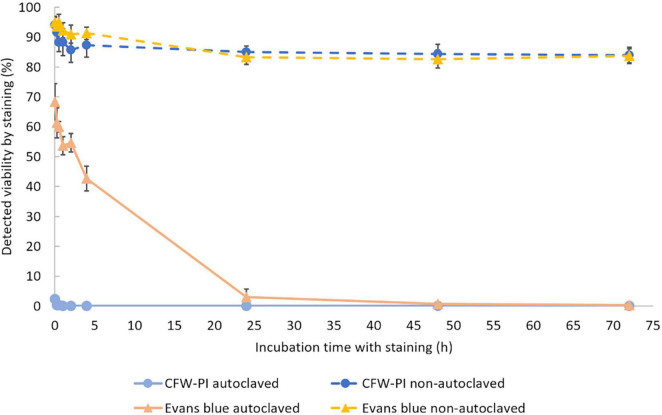
Viability of autoclaved and non-autoclaved spores of *Plasmodiophora brassicae* determined by different staining methods during 72 h of incubation. Error bars indicate standard deviations (*n* = 3).

To determine the optimal incubation time, non-autoclaved spores and autoclaved spores were mixed with Evans blue and CFW-PI, respectively. After 0, 0.25, 0.5, 2, 4, 24, 48, and 72 h of incubation, Evans blue and CFW-PI showed different performances as the incubation time was varied (Figure 4). The viability of autoclaved samples assessed by CFW-PI was 0% and remained stable without changes over time. However, a strong decrease and subsequent stabilization after 24 h of incubation was observed when assessed with Evans blue. For the non-autoclaved samples, the viability detected by Evans blue was slightly higher than that estimated by CFW-PI staining within 4 h incubation. The detected viability remained stable at about 84% with both stainings after 24 h incubation.

**FIGURE 5:**
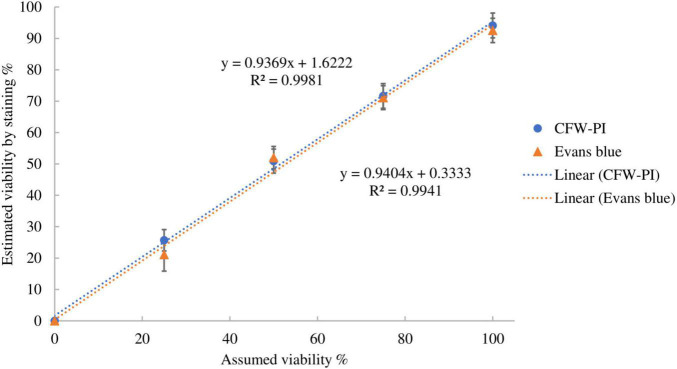
Correlation between assumed viability and estimated viability by staining with Evan blues overnight or CFW-PI for 10 min. Error bars represent standard deviations (*n* = 3).

The accuracy of the two staining methods was evaluated using spore suspensions with known ratios of dead and viable spores by incubating with Evans blue overnight or CFW-PI for 10 min. Figure 5 shows strong positive linear correlations between estimated and assumed viability with coefficients of determination of *R*^2^ = 0.9981 (Evans blue) and *R*^2^ = 0.9941 (CFW-PI), indicating the high accuracy of both staining methods in assessing viability.

### Propidium Monoazide Quantitative PCR Detection of Resting Spore Viability

Standard qPCR cannot distinguish between DNA of viable and dead resting spores. In order to quantify DNA of viable spores, the impact of PMA on inhibiting the amplification of DNA from dead spores was examined.

#### Effect of Lethal Treatments on Resting Spores

**FIGURE 6:**
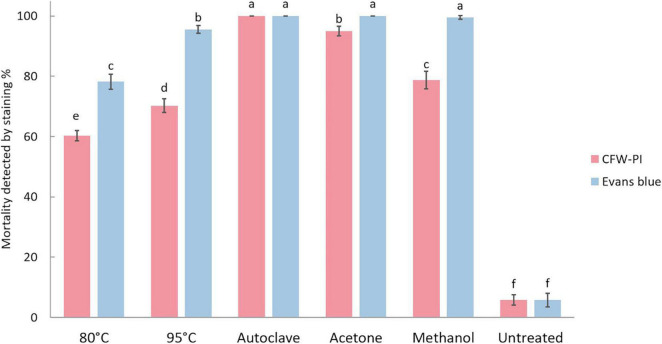
Mortality of *Plasmodiophora brassicae* resting spores assessed by calcofluor white or Evans blue after different lethal treatments. Different letters indicate significant differences among the treatments (Multi-factor ANVOA, Tukey test, *P* < 0.05, *n* = 4). Error bars indicate standard deviations.

The mortality of resting spores was assessed by Evans blue and CFW-PI, which showed significantly higher mortality in the heat and chemical treatments compared to untreated spores (Figure 6). The untreated spores had a mortality of 5.75% determined by both staining methods. Estimating resting spore mortality by incubation with CFW-PI for 10 min showed that the autoclave treatment had the highest mortality of 100%, followed by acetone with 95%. Mortality assessed by overnight incubation with Evans blue showed the highest rate of 100% for autoclave, acetone and methanol treatments, followed by 95°C for 10 min of heat treatment with 95.5% mortality. The mortality of 80°C-1 h treated spores was significantly lower when estimated by CFW-PI (60.25%) than Evans blue (78.25%). For the 95°C-10 min treatment, the mortality predicted by CFW-PI was 35% less than the estimates by Evans blue. For the methanol treatment, the spore mortality was 79% assessed with CFW-PI, whereas it was 100% with Evans blue. In all lethal treatments, the mortality assessed by CFW-PI was significantly lower than Evans blue except for the autoclave treatment.

#### DNA Degradation of Lethal Treatments

**FIGURE 7:**
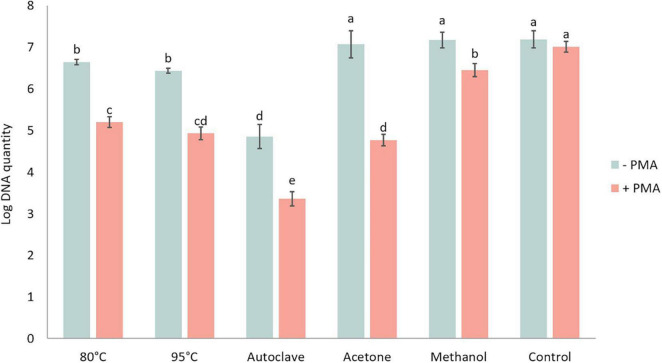
Log DNA amount of *Plasmodiophora brassicae* resting spores quantified by qPCR with (+ PMA) or without (–PMA) propidium monoazide after different heat and chemical treatments.–PMA: total DNA amount; + PMA: DNA of viable spores. Different letters indicate significant differences among the treatments (Multi-factor ANVOA, Tukey test, *P* < 0.05, *n* = 3). Error bars indicate standard deviations.

DNA amount in *P. brassicae* resting spores was quantified in the presence or absence of PMA and showed significant differences between various heat and chemical treatments (Figure 7). The DNA amount in controls with and without PMA was similar, indicating the PMA treatment alone did not affect the quantity of DNA. For the samples without PMA treatment, which was the total DNA amount in each treatment, there was no significant difference in DNA amounts between acetone, methanol and control treatments, while the amount of DNA in heat treatments was significantly lower than in the control treatment. Especially after autoclave treatment, the DNA amount of *P. brassicae* was about 100-fold lower compared to the control. Relative to the total DNA amount, DNA quantified in the presence of PMA was considerably lower. Acetone treatment had the largest decrease when comparing DNA amounts in the same lethal treatment with and without PMA.

#### Accuracy of Propidium Monoazide Quantitative PCR for Assessment of Spore Viability

**FIGURE 8:**
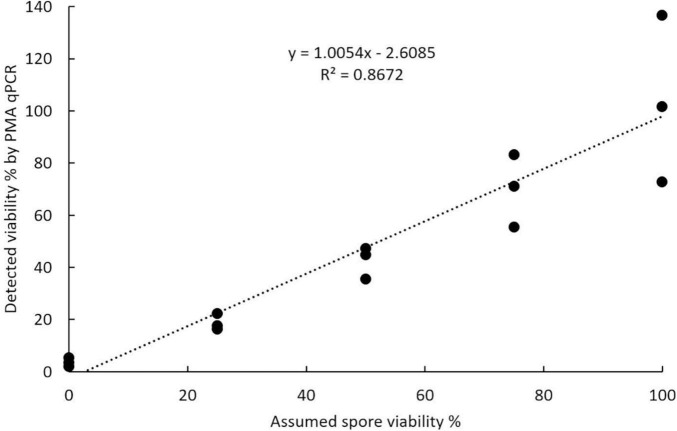
Accuracy of propidium monoazide qPCR in assessing viability calculated using known ratios of viable to acetone-killed resting spores of *Plasmodiophora brassicae*.

The viable and total spore numbers were calculated using the CT value obtained in PMA qPCR to get the viability. Regression analysis of the relationship between the percentage of viable spores detected by PMA qPCR and the assumed percentage of viable spores in the spore suspension showed that these values were positively correlated. The correlation was significant with a *P*-value less than 0.0001 and explained a large percentage of the variance with an *R*^2^ value of 0.8672 (Figure 8).

### Germination Process and Determination of Germination Rate

**FIGURE 9:**
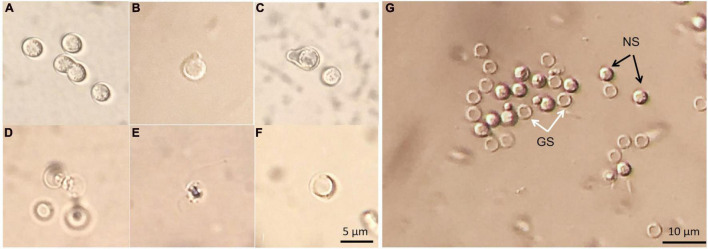
Resting spores of *Plasmodiophora brassicae* under the optical microscope with DIC prism. **(A)** Resting spores and conjugated spores; **(B,C)** emerged “papilla”; **(D)** germinating spore; **(E)** primary zoospore; **(F)** empty case; **(G)** germinated and non-germinated spores, white arrows indicate germinated spores (GS), black arrows indicate non-germinated spores (NS).

The germination process of *P. brassicae* resting spores was observed using light microscopy. There were tiny particles observed in the resting spores that appeared to be in Brownian movement. Occasionally, conjugated spores were observed in spore suspension (Figure 9A). The emergence of “papilla” and the germinating spore that was releasing a primary zoospore were observed (Figures 9B–D). The released zoospore was spherical and biflagellate and the size was similar to a resting spore (Figure 9E). Zoospores swam vigorously at times and less vigorous at others. The observation of zoospores could be disturbed by contaminations, such as some protozoa having similar size and motility. After germination, an empty case with exit pore was left and this was considered as a germinated spore (Figure 9F). The entire process from the emergence of papilla to release of a zoospore in one particular resting spore was difficult to observe. Using DIC microscopy, it was possible to differentiate germinated spores from non-germinated spores as germinated spores showed empty cases and non-germinated spores appeared filled (Figure 9G).

**FIGURE 10:**
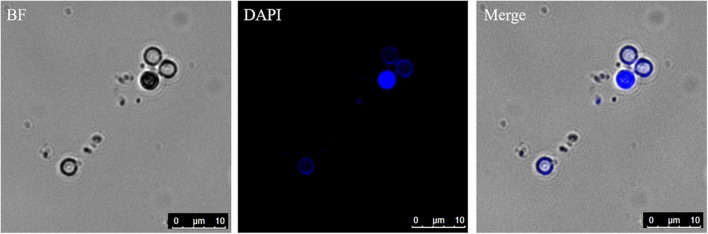
Resting spores stained with DAPI to distinguish the germinated from non-germinated spores with CLSM. The blue spores are non-germinated spores and the colorless spores are germinated spores. BF, bright field.

**FIGURE 11:**
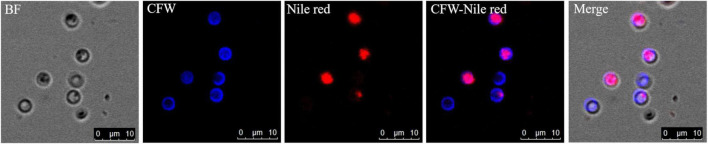
Resting spores of *Plasmodiophora brassicae* stained with the dual staining of CFW-Nile red with confocal microscopy. BF: resting spores under bright field; CFW: cell wall of resting spores stained with calcofluor white; Nile red: lipid droplets stained with Nile red; CFW-Nile red: merge image of dual staining; Merge: merge image of dual staining and bright field (Pink color indicates non-germinated spores, blue color indicates germinated spores).

The germinated spores could be distinguished from non-germinated spores by DAPI and CFW-Nile red staining. The nuclei of non-germinated spores were stained by DAPI and appeared bright blue (Figure 10). Similar to the dual stain of CFW-PI, configuration optimizations were conducted for CFW-Nile red that showed there was no overlap of detectable fluorescence between them. Only the lipid droplets of dead spores were stained by Nile red with an excitation wavelength of 488 nm and an emission wavelength of 590–620 nm. With a lethal pre-treatment, the non-germinated spores, which contained lipid droplets, were stained by both CFW and Nile red, while germinated spores only showed a blue staining of the cell wall (Figure 11).

## Discussion

### Viability Assessment for Resting Spores

To provide powerful tools for life cycle studies of *P. brassicae*, several methods were evaluated for their ability to assess resting spore viability, including fluorescein diacetate, trypan blue, methylene blue, acridine orange (data not shown), Evans blue and CFW-PI as well as PMA qPCR. After several comparisons, we found that Evans blue and CFW-PI were the most reliable staining methods for determining resting spore viability. Evans blue has been applied for evaluating *P. brassicae* resting spore viability by Tanaka et al. (1999). However, Al-Daoud et al. (2017) proposed that the estimated viability obtained by incubating for 30 min with Evans blue was inaccurate. Our studies showed that the viability of *P. brassicae* resting spores estimated by Evans blue became reliable only after 24 h of incubation (Figure 4), which was consistent with Harding et al. (2019) reporting that a longer exposure time (8 h or more) was able to improve the accuracy and consistency of evaluation. Insufficient incubation time of less than 4 h lead to an underestimation of mortality by 40%, introducing experimental error. Evans blue showed no significant difference of estimates when the exposure time was longer than 24 h, suggesting that it did not have toxic effects on the spores during 72 h of incubation. Therefore, overnight incubation is applicable and convenient for Evans blue when used to assess the viability of resting spores.

In addition, fluorescence microscopy using the cellulose and chitin binding fluorochrome CFW has been employed in combination with the mutagenic nucleic acid stain ethidium bromide (EB) to assess the viability of *P. brassicae* resting spores (Takahashi and Yamaguchi, 1988; Takahashi, 1991; Donald et al., 2002). Due to the high health risks of EB, in our study, the cell-membrane impermeable dye PI was selected as an alternative that binds to nucleic acids by intercalating between the bases with little sequence preference. It has been reported that 30 μM of PI was cell permeable and cytotoxic to a macrophage cell line (J774 cells) after 1 day of incubation (Chiaraviglio and Kirby, 2014). A concentration of 10 μM PI was able to facilitate partial PI penetration resulting in potential false positive results (Krämer et al., 2016). In the present study, a final concentration of 3 μM (2 μg/ml) PI was used in the *P. brassicae* spore suspension. The dual staining with CFW-PI showed a clear differentiation between viable and non-viable spores (Figure 3). CFW-PI provided stable viability with prolonged exposure of 72 h, suggesting that 3 μM PI was applicable as it did not cause cytotoxicity or excessive permeability to the spores. In addition, dual staining with CFW-PI was capable of providing reliable predictions of spore viability within 10 min, which was much faster than Evans blue. Strong positive linear correlations between predicted and assumed viability by CFW-PI and Evans blue revealed that the accuracy of both staining methods was more than 99%, suggesting that they can be applied for estimating the viability of *P. brassicae* resting spores.

After optimization, the staining methods of CFW-PI and Evans blue were applied for evaluating the lethal effects of heat and chemical treatments on resting spores. Among the treatments, autoclaved spores had the highest mortality assessed by both staining methods. For each heat and chemical treatment, there were significant differences in the viability estimated by CFW-PI and Evans blue which could be explained by the difference in the staining duration between the two methods (Figure 6). The treated spores stained with CFW-PI were evaluated within 10 min, showing the instant viability after the lethal treatments. However, the spores stained with Evans blue were assessed after overnight incubation, which was the final viability of each lethal treatment. After overnight incubation, the mortality rate increased significantly, meaning that there was a delay in the death of the spores after lethal treatments. Perhaps, the speed (or accessibility) of staining also depended on the effect of the treatments on the degree of physical destruction of the spores, e.g., mortality increased with increasing temperatures.

Propidium monoazide is a cell membrane impermeable dye that photoreactively binds to double-stranded DNA (dsDNA) with high affinity. Upon photolysis, it selectively modifies DNA from dead cells with compromised membrane integrity, while DNA from living cells is not affected. Based on this mechanism PMA qPCR has been applied for the quantification of viable cell DNA as an estimate of the rate of viable cells (Nocker et al., 2006, 2007a,b).

Several studies have attempted to use heat-killed *P. brassicae* resting spores to evaluate the accuracy of PMA qPCR (Al-Daoud et al., 2017; Harding et al., 2019). However, our results showed that DNA integrity would be affected by heat treatments but not by chemical treatments, such as acetone and methanol. This damage effect of heat treatments may have a strong impact on the evaluation of PMA qPCR accuracy. Therefore, chemical treatments, especially acetone, are recommended for such studies with artificially induced lethality of spores to construct standard spore suspension series. A strong linear correlation between detected viability and assumed viability based on plasmid DNA, suggesting that PMA qPCR can be used to detect the viability of resting spores, but the obtained results were more variable than the staining methods.

Among all tested dyes, Evans blue and CFW-PI staining are the feasible and accessible approaches to determine *P. brassicae* resting spore viability. While Evans blue requires overnight incubation to obtain stable viability, the application of CFW-PI allows the detection of viability precisely and quickly. PMA-qPCR is another option that can be applied for the samples which cannot be determined by staining methods, such as soil samples. Thus, our studies provide multiple options for assessing the viability of resting spores that can be employed for various life cycle studies.

### Germination of Resting Spores and Assessment of Germination Rates

Resilient *P. brassicae* resting spores containing chitin in cell walls can survive in the soil for long periods and will germinate to release zoospores when the conditions are favorable. The emergence of papilla was the first sign of germination. Each spore released one primary zoospore, which is required to infect plant tissue. Germinated spores showed empty cases without any content. The observed germination process in the present study was consistent with the observation by Macfarlane (1970). However, until today, no one has observed the entire process from emergence of papilla to release of a zoospore in one particular resting spore. Hence, it is still unclear how long the whole germination process takes and whether it is a slow progressive or instantaneous process. The movement patterns of zoospores also remain unknown by now. The small size of resting spores (ca. 3 μm) and microbial contaminations in spore suspensions impede a competent and high-quality observation. Live cell imaging using time-lapse microscopy with high resolution may be a good option for long-term recording of the germination process.

Primary zoospores are considered as the main source of infection, but they are not easy to be directly counted. The presence of empty spores provides good evidence of germination. The clear distinction between germinated and non-germinated spores is essential for evaluating the effects of germination stimulating substances, which may have the potential to control the disease. Several methods have been used to distinguish germinated from non-germinated spores in previous studies, such as orcein (Friberg et al., 2005; Rashid et al., 2013). However, orcein did not perform very well in our tests as only a part of the spores was stained rather than all the spores containing nuclei, indicating orcein could not provide reliable estimates of germination rates (data not shown). A blue fluorescent nucleic acid stain, DAPI, has been used to determine the germination of resting spores by the absence of nuclei of spores (Niwa et al., 2008). DAPI forms fluorescent complexes with AT clusters having one molecule of dye per 3 base pairs (Kapuściński and Szer, 1979). Clear images of *P. brassicae* nuclei stained with DAPI were obtained (Figure 10). The dual staining of CFW combined with the lipophilic fluorescent dye Nile red has been employed for examining the structures and development of *P. brassicae* in *B. napus* callus tissues (Tu et al., 2019). Nile red has also been used to stain intracellular lipid droplets abundant in resting spores of *P. brassicae* (Bi et al., 2016). The absence of lipid droplets could also be considered as an indicator of discriminating between germinated and non-germinated spores. However, we found that Nile red was only able to stain the lipid droplets of non-viable resting spores rather than all the spores containing lipids. This may be attributed to the excessive thickness of the cell wall of the mature spores that affected the intrusion of the dye (Rumin et al., 2015). CFW binding to cellulose and chitin was used to label the cell walls of all *P. brassicae* resting spores. Therefore, CFW-Nile red could be applied to distinguish the germinated spores from non-germinated spores with a prerequisite to inactivate the spores for successful staining (Figure 11). Differential interference contrast microscopy is a fast and easy to use method that has been employed in several studies (Naiki et al., 1987; Suzuki et al., 1992; Asano et al., 2000), which were consistent with our results that germinated spores showed empty cases and non-germinated appeared filled (Figure 9).

Taken together, this study presents reliable methodological tools essential to study the factors in soil regulating survival, dormancy and germination of *P. brassicae* resting spores as the key life cycle stages inciting infection and disease in Brassicaceae hosts. Thus, these tools may enable an advanced understanding of factors in the plant-soil-pathogen interface leading to damage by clubroot and allow for the design of novel strategies to manage this disease.

## Data Availability Statement

The raw data supporting the conclusions of this article will be made available by the authors, without undue reservation.

## Author Contributions

YW designed, performed all the experiments, and wrote the manuscript. BK was involved in constructing samples and advising. AT provided project guidance and revised the manuscript. All authors contributed to the manuscript and approved the submitted version.

## Conflict of Interest

The authors declare that the research was conducted in the absence of any commercial or financial relationships that could be construed as a potential conflict of interest.

## Publisher’s Note

All claims expressed in this article are solely those of the authors and do not necessarily represent those of their affiliated organizations, or those of the publisher, the editors and the reviewers. Any product that may be evaluated in this article, or claim that may be made by its manufacturer, is not guaranteed or endorsed by the publisher.
